# Supplementary material for: DMRforPairs: identifying Differentially Methylated Regions between unique samples using array based methylation profiles
Source: BMC Bioinformatics. 2014 May 15;15:141. doi: 10.1186/1471-2105-15-141 (PMC4046028; doi:10.1186/1471-2105-15-141)
Supplement: Additional file 2 — DMRforPairs output for the comparison of A431-MCF7 and NA17018-NA17105. Please start from the HTML files in each folder. Available via the BMC Bioinformatics website. [file 1471-2105-15-141-S2.zip › 1394847754114233_MOESM2_ESM/A431_MCF7/figures/31821.pdf]

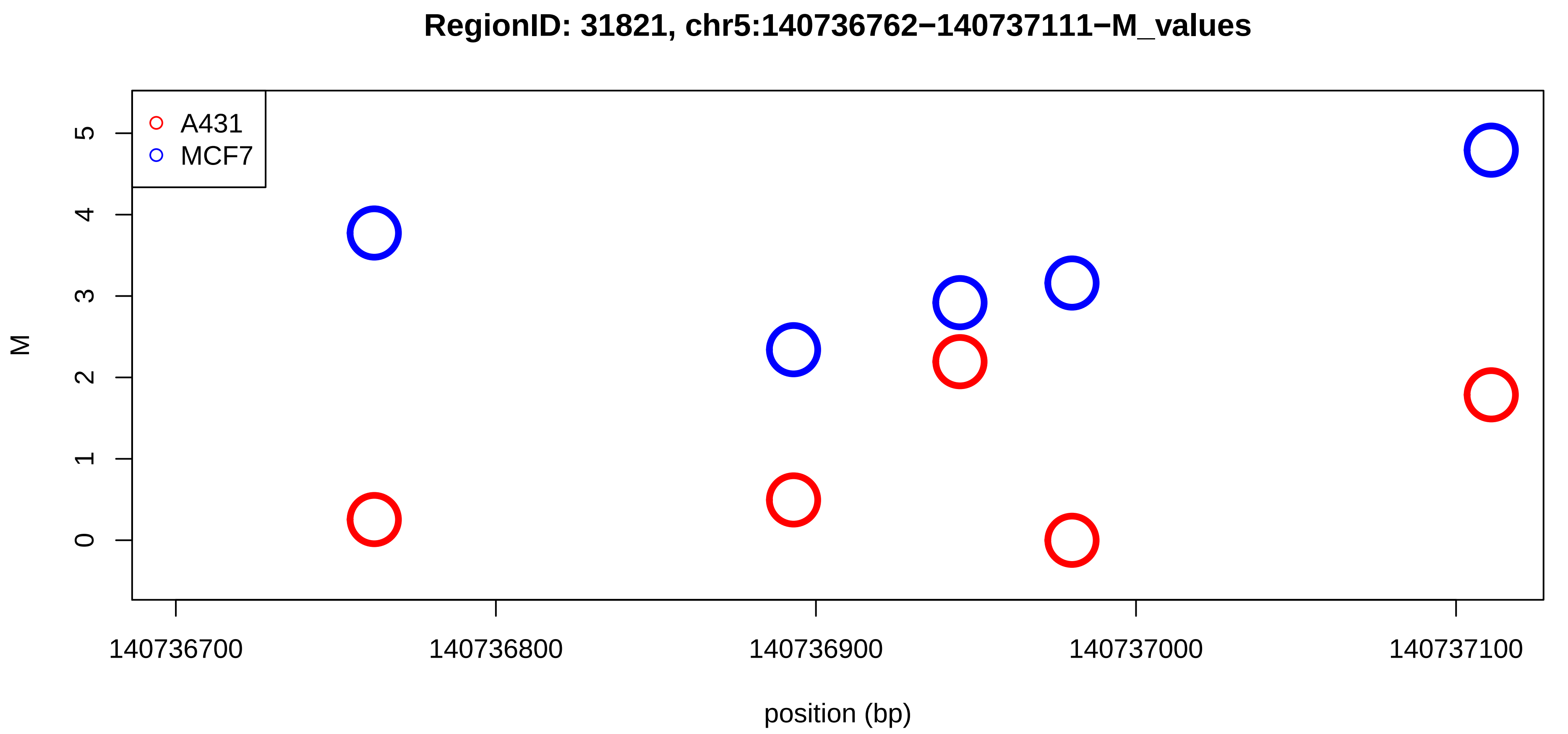
RegionID: 31821, chr5:140736762−140737111−M_values
A431
MCF7
M
0
1
2
3
4
5
140736700
140736800
140736900
140737000
140737100
position (bp)

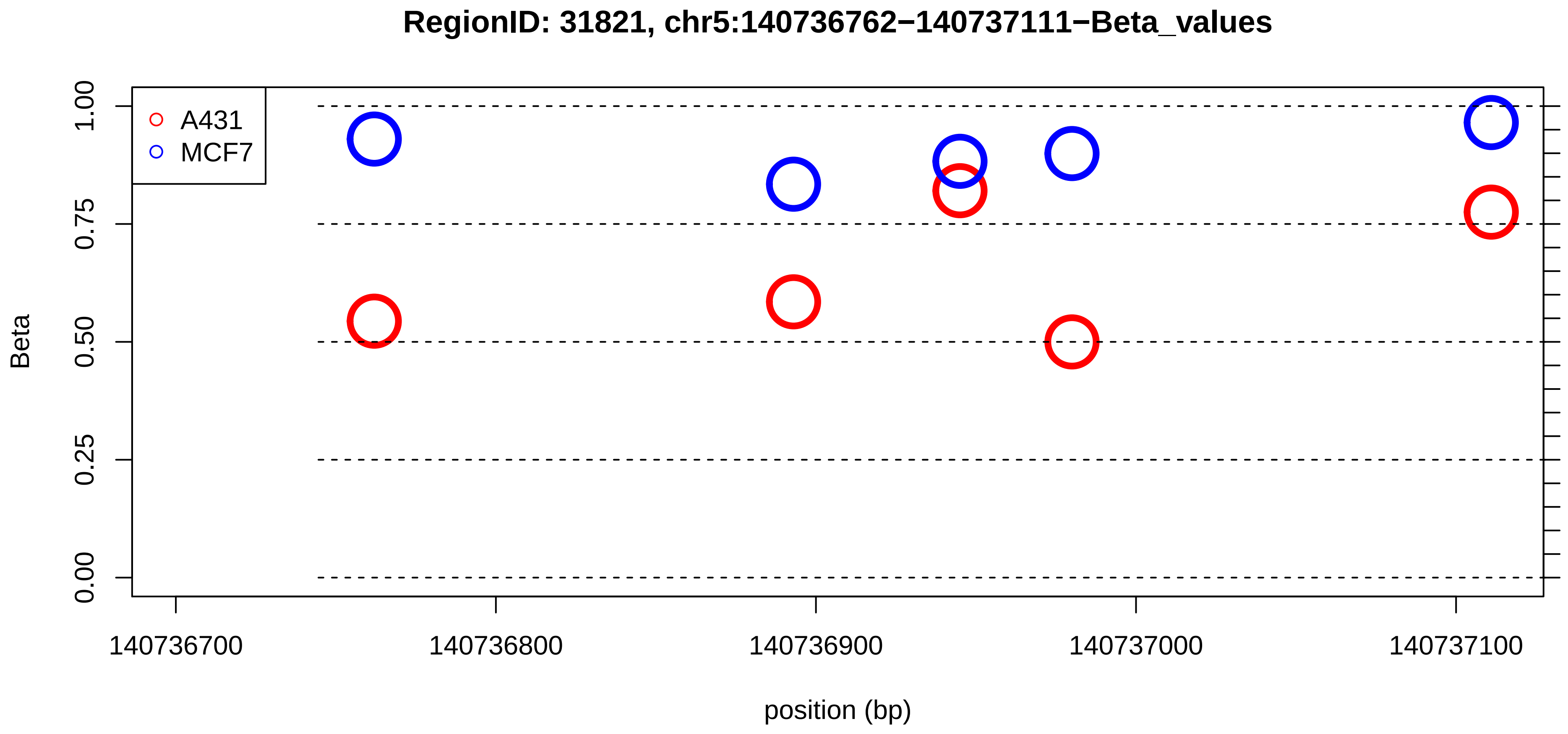
RegionID: 31821, chr5:140736762−140737111−Beta_values
A431
MCF7
Beta
0.00
0.25
0.50
0.75
1.00
140736700
140736800
140736900
140737000
140737100
position (bp)

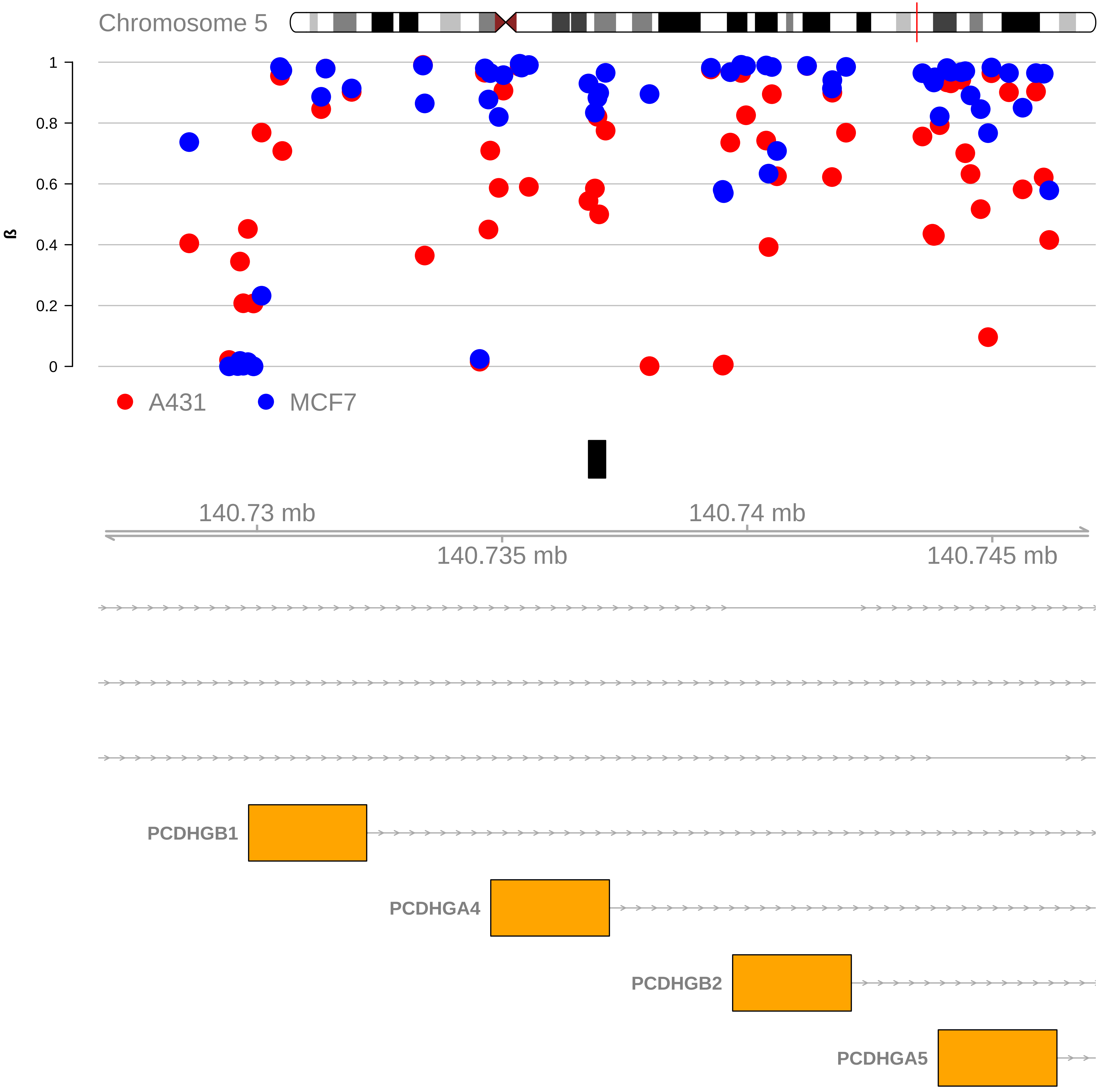

Chromosome 5
1
0.8
0.6
0.4
0.2
0
ß
A431
MCF7
140.73 mb
140.74 mb
140.735 mb
140.745 mb
PCDHGB1
PCDHGA4
PCDHGB2
PCDHGA5
